# St. Louis Medical Society and the Revision of the Code of Ethics

**Published:** 1883-10

**Authors:** 


### THE ST. LOUIS MEDICAL SOCIETY AND THE REVISION OF THE CODE OF ETHICS.

It wlil be remembered that at the late meeting of the American Medical Association, Dr. Pollak, of St. Louis, on behalf of the St. Louis Medical Society, moved the appointment of a committee to revise the Code of Ethics, which motion was promptly laid on the table. It now appears that this resolution was not authorized by the St. Louis Medical Society, and that Dr. Pollak presented it without consulting his brother delegates. At a late meeting, the St. Louis Medical Society repudiated and condemned the action of Dr. Pollak, with only two dissenting votes. The St. Louis Medical Society sits down upon Dr. Pollak, in this manner; On June 23, 1883, Dr. Atwood introduced the following, which the St. Louis Society adopted after some discussion :

WHEREAS, At the recent session of the American Medical Association, a preamble and resolution were offered for the consideration of said Association, purporting to represent the sense of the St. Louis Medical Society upon the propriety of preparing a new Code of Ethics, or altering and changing the existing code in accordance with the present relations of the profession ; and

WHEREAS, In said preamble the assertion is made that "the Code has accomplished all it was designed it should, but at present many of its features are obsolete and not adapted to our wants. The necessity of an early revision is very apparent, is loudly called for in all parts of our land, and cannot be repressed much longer. . . . The time has come when the loud and very soon universal call will have to be heeded ;" and

WHEREAS, The St. Louis Medical Society did not instruct, "That the committee be authorized to prepare a Code of Ethics which in their view will meet the wishes of the profession, and submit the same to the meeting of 1884 ;" therefore

*Resolved*, That the St. Louis Medical Society distinctly repudiates the statements contained in said preamble, and again expresses its fealty to the existing Code of Ethics as a time-honored and most suitable fundamental law of the profession, and specially deprecates any action calculated to reflect upon its loyalty to those principles which have heretofore secured immunity from the machinations of schismatics within or enemies without.—*Louisville Medical News*.

